# Reviewer acknowledgement 2014

**DOI:** 10.1186/s12879-015-0749-7

**Published:** 2015-01-29

**Authors:** Philippa K Harris

**Affiliations:** BioMed Central, 236 Gray’s Inn Road, London, WC1X 8HB UK

## Abstract

The editors of *BMC Infectious Diseases* would like to thank all our reviewers who have contributed to the journal in Volume 14 (2014).

**Jan Aarnoudse**

Netherlands

**Raquel Abad**

Spain

**Emmanuel Abatih**

Belgium

**Penelope Abbott**

Australia

**Atqah Abdul Wahab**

Qatar

**Fekadu Abebe**

Norway

**Delbert Abi Abdallah**

USA

**Asha Abraham**

India

**Richard Adegbola**

Belgium

**Ayola Akim Adegnika**

Gabon

**Jeanne Adiwinata Pawitan**

Indonesia

**Yaw Afrane**

Kenya

**Vishnu Agarwal**

India

**Oche Agbaji**

Nigeria

**Anju Aggarwal**

India

**Rakesh Aggarwal**

India

**Ashutosh Aggarwal**

India

**Anurag Agrawal**

India

**Fabio Aguiar-Alves**

Brazil

**Golo Ahlenstiel**

Australia

**Niyaz Ahmed**

India

**Sunil Ahuja**

USA

**Peter Aka**

USA

**Sally Akarolo-Anthony**

USA

**Karolina Akinosoglou**

Greece

**Eleni Aklillu**

Sweden

**Chantal Akoua-Koffi**

Cote d’Ivoire

**Sercan Aksoy**

Turkey

**Adam Akullian**

USA

**Mohamed Al-Agamy**

Saudi Arabia

**Sophie Alain**

France

**Alhadi Alamir**

Egypt

**Amr Albanna**

Saudi Arabia

**Catharina Johanna Alberts**

Netherlands

**Marco Albonico**

Italy

**Stefano Alcaro**

Italy

**Mercedes Alemán**

Argentina

**Bryan Alexander**

USA

**Vangelis Alexiou**

Greece

**Nahid Ali**

India

**Jean-Pierre Allain**

UK

**Franz Allerberger**

Austria

**Benito Almirante**

Spain

**Nina Alphey**

UK

**Zubaida Daham Al-Suwaidi**

Qatar

**Tugrul Altan**

Turkey

**Ben Althouse**

USA

**Mario Alvarez**

Mexico

**Gerardo Alvarez-Uria**

India

**Deepak Amarapurkar**

India

**Massimo Amicosante**

Italy

**Janaki Amin**

Australia

**Roland Ammann**

Switzerland

**Joshua Amo-Adjei**

Ghana

**Zhijie An**

China

**Devasena Anantharaman**

France

**Suzanne Anderson**

Gambia

**Sören Andersson**

Sweden

**Jon Kim Andrus**

USA

**Irene Ang**

Hong Kong

**Mehar Angez**

Pakistan

**Francois Angoulvant**

France

**Fernanda Anibal**

Brazil

**Spinello Antinori**

Italy

**Henedina Antunes**

Portugal

**Balakrishnan Anukumar**

India

**Tetsuji Aoyagi**

Japan

**Victor H Aquino**

Brazil

**Michael Arabatzis**

Greece

**Sophia Archuleta**

Singapore

**Karim Asehnoune**

France

**Angel Asensio**

Spain

**Elizabeth Ashley**

Thailand

**Ruth Ashton**

Uganda

**Ulysse Ateba Ngoa**

Gabon

**Etienne Audureau**

France

**Sara Auld**

USA

**Linda Aurpibul**

Thailand

**Robin Avery**

USA

**Cenk Aypak**

Turkey

**Mehdi Azami**

Iran

**Andrew Azman**

USA

**Eduardo Azziz-Baumgartner**

USA

**Subash Babu**

India

**Sandra Baffour-Awuah**

Ghana

**Patrizia Bagnarelli**

Italy

**Sabrina Bakeera-Kitaka**

Uganda

**Manju Bala**

India

**Vincenzo Baldo**

Italy

**Vibe Cecilie Ballegaard**

Denmark

**David Banach**

USA

**Pamela Barbadoro**

Italy

**Christovam Barcellos**

Brazil

**Francesco Barchiesi**

Italy

**Olivia Bargiacchi**

Italy

**Shams Bari**

India

**Timothy Barkham**

Singapore

**Gavin Barlow**

UK

**Dale Barnard**

USA

**Rosana Barros**

Brazil

**Luisa Barzon**

Italy

**Carol Bassim**

USA

**Monica Basso**

Italy

**Nicole Basta**

USA

**Mathieu Bastard**

France

**Alan Bates**

UK

**Matthew Bates**

UK

**Abebe Genetu Bayih**

Canada

**Orhan Baylan**

Turkey

**Roger Bayston**

UK

**Frank Beard**

Australia

**Nicholas Beare**

UK

**Pascal Beaudeau**

France

**Cecile Bebear**

France

**Ingeborg Becker**

Mexico

**Sarah Beckham**

USA

**Simon Beddows**

UK

**Rodolfo Begue**

USA

**Deepak Behera**

USA

**Joachim Beige**

Germany

**Ulla Beijer**

Sweden

**Yeshambel Belyhun**

Ethiopia

**Ronen Ben-Ami**

Israel

**Andrea Benedetti**

Cape Verde

**Natividad Benito**

Spain

**Constance Benson**

USA

**Dennis Bente**

USA

**Juan Berenguer**

Spain

**Thomas Berg**

Germany

**Peter Bergman**

Sweden

**Dag Berild**

Norway

**Johannes Berkhof**

Netherlands

**James Berkley**

Kenya

**Guyah Bernard**

Kenya

**Louis Bernard**

France

**Jose Ignacio Bernardino**

Spain

**David Berrigan**

USA

**William Berrington**

USA

**Matthew Berry**

UK

**Giovanna Bertini**

Italy

**Khalid Beshir**

UK

**John Best**

USA

**Victoria Best**

USA

**Renu Bharadwaj**

India

**Sujit Bhattacharya**

India

**Samit Bhattacharyya**

USA

**Federico Biagi**

Italy

**Tiffany Bias**

USA

**Matthew Biggerstaff**

USA

**Alberto Biglino**

Italy

**Efraim Bilavsky**

Israel

**Tannaz Birdi**

India

**Andre Birgy**

France

**Marius Birlea**

USA

**Zeno Bisoffi**

Italy

**Jim Black**

Australia

**Stuart Blacksell**

Thailand

**Laurent Blairon**

Belgium

**Jorge Blanco**

Spain

**Jose M Blasco**

Spain

**Ana Blas-Garcia**

Spain

**Lucie Blok**

Netherlands

**Koen Blot**

Belgium

**Stijn Blot**

Belgium

**Daniel Boateng**

Ghana

**Sara Boccalini**

Italy

**Thomas Bock**

Germany

**Pierre-Yves Boelle**

France

**Sandra Boiça Silva**

Brazil

**Nicholas Boire**

USA

**Jesper Bonde**

Denmark

**Maryline Bonnet**

Switzerland

**Andre Boonstra**

Netherlands

**Ken Boorom**

USA

**Robert Booth**

USA

**Jose Bordon**

USA

**Alberto Borghetti**

Italy

**Victor Borja-Aburto**

Mexico

**Anna Boron-Kaczmarska**

Poland

**Maria-José Borrego**

Portugal

**Franco Borruto**

Monaco

**Aik Bossink**

Netherlands

**John Bosso**

USA

**Elisabeth Botelho-Nevers**

France

**Graham Bothamley**

UK

**Julie Bottero**

France

**Claire Bourke**

UK

**Emilio Bouza**

Spain

**Scott Bowden**

Australia

**Bulent Bozdogan**

Turkey

**Janette Bradley**

UK

**Daniel Bradshaw**

Australia

**Paula Braitstein**

USA

**Rachel Bras-Gonçalves**

France

**Reginaldo**

Brazil

**Patrick Brennan**

USA

**Silvia Bressan**

Italy

**Richard Brilli**

USA

**Sylvain Brisse**

France

**William Britt**

USA

**Susanne Brix**

Denmark

**Susan Brogly**

USA

**James Brooks**

Canada

**Shobha Broor**

India

**Florence Brossier**

France

**Michael Brown**

UK

**Michael Browning**

UK

**Enrico Brunetti**

Italy

**Bianca Bruzzone**

Italy

**Filemon Bucardo**

Nicaragua

**Udo Buchholz**

Germany

**Bryce Buddle**

New Zealand

**Maria Jose Buitrago**

Spain

**David Burger**

Netherlands

**Patrick Butaye**

Belgium

**Andrea Maurizio Cabibbe**

Italy

**Alida Caforio**

Italy

**Rui Cai**

Netherlands

**Dawei Cai**

USA

**Morena Caira**

Italy

**Andrea Calcagno**

Italy

**Maire Callaghan**

Ireland

**Annapaola Callegaro**

Italy

**Ricardo Jorge Camacho**

Belgium

**Calogero Cammà**

Italy

**Floriana Campanile**

Italy

**Monica Campo**

USA

**Cláudio Canal**

Brazil

**Ellen Caniglia**

USA

**Chunxiang Cao**

China

**Bin Cao**

China

**Jacqueline Capeau**

France

**Gioia Capelli**

Italy

**Xavier Carcopino**

France

**Luciana Cardoso**

Brazil

**Dianne Carey**

Australia

**Mauricio Carobene**

Argentina

**Fabrice Carrat**

France

**Margaret Carrel**

USA

**Patrizia Carrieri**

France

**Maimuna Carrim**

South Africa

**Enitan Carrol**

UK

**Michael Carter**

UK

**Edgar M. Carvalho**

Brazil

**Peg Carver**

USA

**Beatrice Casini**

Italy

**Edana Cassol**

USA

**Vicente Catalan**

Spain

**Lucas Cavallaro**

Italy

**Maxine Caws**

Viet Nam

**Dayaraj Cecilia**

India

**Carina Cesar**

Argentina

**Anis Chaari**

Tunisia

**Dave D. Chadee**

Trinidad and Tobago

**Vineet Chadha**

India

**Ann Chahroudi**

USA

**Shua Chai**

USA

**Catriona Chalmers**

UK

**Rachel Chalmers**

UK

**Steve Chambers**

New Zealand

**Karen Champenois**

France

**Albert Chan**

Hong Kong

**Yoke-Fun Chan**

Malaysia

**Shuk-Ying Chan**

Hong Kong

**K H Chan**

Hong Kong

**Monica Chan**

Singapore

**Thomas Sy Chan**

Hong Kong

**Duncan Chanda**

Zambia

**Feng-Yee Chang**

Taiwan

**Sui-Yuan Chang**

Taiwan

**Bai Changqing**

China

**Soranun Chantarangsu**

Thailand

**Ilias Chantziaras**

Greece

**Mei Chao**

Taiwan

**Patrick Charles**

Australia

**Charlotte Charpentier**

France

**Mitali Chatterjee**

India

**Stylianos Chatzipanagiotou**

Greece

**Kimon Chatzistamatiou**

Greece

**Abhijit Chaudhuri**

UK

**Sidhartha Chaudhury**

USA

**Kalyan Chavda**

USA

**Luis Fernando Chaves**

Japan

**Po-Lin Chen**

Taiwan

**Liang Chen**

USA

**Chien-Yi Chen**

Taiwan

**Qijun Chen**

China

**Chien-Chou Chen**

Taiwan

**You-Hua Chen**

Canada

**Yen-Hsu Chen**

Taiwan

**Jiazhen Chen**

China

**Jonathan Chen**

Hong Kong

**Ji Yih Chen**

Taiwan

**Luke Chen**

USA

**Marcus Chen**

Australia

**Ray Chen**

USA

**Szu Chieh Chen**

Taiwan

**Pin-Nan Cheng**

Taiwan

**Vincent CC Cheng**

Hong Kong

**Jason Cheung**

Hong Kong

**Ramsey Cheung**

USA

**Stephane Chevaliez**

France

**Mala Chhabra**

India

**Antonio Chiaretti**

Italy

**Thaweesak Chieochansin**

Thailand

**Erica Chimara**

Brazil

**Natsayi Zanile Chimbindi**

South Africa

**Wei-Mei Ching**

USA

**James Chipeta**

Zambia

**Joconiah Chirenda**

Zimbabwe

**Cheng-Hsun Chiu**

Taiwan

**Nan-Chang Chiu**

Taiwan

**Seung-Hak Cho**

South Korea

**Gabriel Chodick**

Israel

**Siu Ho Chok**

Hong Kong

**Kulkanya Chokephaibulkit**

Thailand

**Chia Yin Chong**

Singapore

**Pele Chong**

Taiwan

**Eric Chow**

Australia

**Gerardo Chowell**

USA

**Ana Marli Christovam Sartori**

Brazil

**Karen Chronister**

Australia

**Yiu Wai Chu**

Hong Kong

**Kyra Chua**

Australia

**Jen-Hsiang Chuang**

Taiwan

**Abrar Chughtai**

Australia

**Shiu-Dong Chung**

Taiwan

**Catia Cilloniz**

Spain

**Mareli Claassens**

South Africa

**Jan Clement**

Belgium

**Christopher John Clements**

Australia

**Gary Clifford**

France

**Ahmet Yilmaz Coban**

Turkey

**Adam Cohen**

South Africa

**Elisabeth Cohen**

USA

**David Cohn**

USA

**Susan Cohn**

USA

**Michelle Cole**

UK

**Julio Collazos**

Spain

**Claudia Colomba**

Italy

**Rhonda Colombo**

USA

**Felipe J. Colon Gonzalez**

Italy

**Vivian Colon-Lopez**

Puerto Rico

**Maurizio Comanducci**

Italy

**Stephen Conaty**

Australia

**Marcus Conde**

Brazil

**Maria Pia Conte**

Italy

**Paul Converse**

USA

**Geoffrey Coombs**

Australia

**Keith Cooper**

UK

**Lawrence Copelovitch**

USA

**Nicola Coppola**

Italy

**Marli Cordeiro**

Brazil

**Anne Cori**

UK

**Alyssa Cornall**

Australia

**Dolores Correa**

Mexico

**Paul Corstjens**

Netherlands

**Sofia Cortes**

Portugal

**Andrea Cossarizza**

Italy

**Anna-Maria Costa**

Australia

**Cristina Costa**

Italy

**Sylvia Costa**

USA

**Claudio Costantino**

Italy

**Jean Tenena Coulibaly**

Cote d’Ivoire

**Benjamin Cowling**

Hong Kong

**Philip Craig**

UK

**Lisa Cranmer**

USA

**Inma Crespo**

Spain

**Francesco Cristini**

Italy

**Pablo Cruces**

Chile

**Nancy Crum-Cianflone**

USA

**Andrea Cruz**

USA

**Isra Cruz**

Spain

**Lunbiao Cui**

China

**Burke Cunha**

USA

**Aubrey Cunnington**

UK

**Bryan Curtin**

USA

**Maria Grazia Cusi**

Italy

**Felicity Cutts**

France

**Lucette Cysique**

Australia

**Christopher Czaja**

USA

**Mustafa Dabbous**

USA

**Ritwik Dahake**

India

**Paulo De Tarso Dalcin**

Brazil

**Stephanie Dancer**

UK

**M. Carolina Danovaro-Holliday**

USA

**Larry Danziger**

USA

**Rupak Datta**

USA

**Regina Daumas**

Brazil

**Michael David**

USA

**Mark Davies**

Australia

**Alissa Davis**

USA

**Luke Davis**

USA

**Meghan Davis**

USA

**Rebecca Davis**

Australia

**Mohamed Ali Daw**

Libya

**Halima Dawood**

South Africa

**Peter Dawson**

Australia

**Carolyn Day**

Australia

**Ghassan Dbaibo**

Lebanon

**Elena De Carolis**

Italy

**Liane De Castro**

Brazil

**Elizabeth De Gaspari**

Brazil

**Bouke De Jong**

Belgium

**Adelzon De Paula**

Brazil

**Alexandre De Paula Rogerio**

Brazil

**Annebel De Vleeschauwer**

Belgium

**Jan De Waele**

Belgium

**Anna Dean**

Switzerland

**Anne Deckert**

Canada

**Martin Dedicoat**

UK

**Huang Dedong**

China

**Abraham Degarege**

Ethiopia

**Takashi Deguchi**

Japan

**Francois Delahaye**

France

**Eugene Dempsey**

Ireland

**Ian Denham**

Australia

**Pieter Depuydt**

Belgium

**Olufemi Desalu**

Nigeria

**Jean-Claude Desenclos**

France

**Jagadish Deshpande**

India

**Sophie Desmonde**

France

**Ulrich Desselberger**

UK

**Roger Detels**

USA

**Gabriella D’Ettorre**

Italy

**Sylvie Deuffic-Burban**

France

**Gregor Devine**

Australia

**Brecht Devleesschauwer**

Belgium

**Subramanian Dhandayuthapani**

USA

**Keertan Dheda**

South Africa

**Mehul Dhorda**

France

**Ilaria Di Bartolo**

Italy

**Simona Di Giambenedetto**

Italy

**Robert Dickson**

USA

**Javier Diez-Domingo**

Spain

**Joakim Dillner**

Sweden

**Jo-Anne R Dillon**

Canada

**Andrew Dinardo**

USA

**Trung Dinh**

The Viet Nam

**Brent Dixon**

Canada

**Pete Dodd**

UK

**Sam Doerken**

Germany

**Pascal Dohmen**

Germany

**Pere Domingo**

Spain

**Massimiliano Don**

Italy

**Peter Donald**

South Africa

**Tao Dong**

UK

**Domenica Donia**

Italy

**Ruben Donis**

USA

**Tjibbe Donker**

Netherlands

**Carmen Mihaela Dorobat**

Romania

**Monika Dos Santos**

South Africa

**Horng-Yunn Dou**

Taiwan

**David Dowdy**

USA

**Mike Drebot**

Canada

**Richard Drew**

Ireland

**Steven Drews**

Canada

**Maria Drogari-Apiranthitou**

Greece

**Eric Druyts**

Canada

**Matthew Dryden**

UK

**Hong Du**

USA

**Ning Du**

USA

**Mignon Du Plessis**

South Africa

**Anuradha Dube**

India

**Luiz Duczmal**

Brazil

**Anne Sophie Dugast**

USA

**Nicole Dukers-Muijrers**

Netherlands

**Herve Dupont**

France

**Nizam Duran**

Turkey

**Paolo Durando**

Italy

**Emanuele Durante Mangoni**

Italy

**Ravi Dyavar**

USA

**Chadli Dziri**

Tunisia

**Kirstine Eastick**

UK

**Jose Echevarria**

Spain

**Miriam Eddyani**

Belgium

**E. Jennifer Edelman**

USA

**Paul Edelson**

USA

**Andrew Edmonds**

USA

**Andrew Edwards**

UK

**Kathryn Edwards**

USA

**Ochaka Egesie**

Nigeria

**Philippe Eggimann**

Switzerland

**Beverly Egyir**

Ghana

**Jan Ehrchen**

Germany

**Boris P Ehrenstein**

Germany

**Thomas Eisele**

USA

**Anna Eis-Huebinger**

Germany

**Zelal Ekinci**

Turkey

**Koumavi Didier Ekouevi**

Togo

**Wael Elamin**

Ireland

**Descloux Elodie**

New Caledonia

**A. Rani Elwy**

USA

**Katherine Emary**

UK

**Christopher Emery**

USA

**Andrea Endimiani**

Switzerland

**Hock-Liew Eng**

Taiwan

**Martin Johannes Enk**

Brazil

**Koray Ergunay**

Turkey

**Kristine Erlandson**

USA

**Vanessa Escuret**

France

**Claudia Estcourt**

UK

**Angel Estella**

Spain

**Chris Estes**

USA

**Vicente Estrada**

Spain

**Gilles Etienne**

France

**Magnus Evander**

Sweden

**Charlesnika Evans**

USA

**Joseph Eyo**

Nigeria

**Massimiliano Fabbiani**

Italy

**Meredith Faires**

Canada

**Christopher Fairley**

Australia

**Marco Falcone**

Italy

**Giacomo Faldella**

Italy

**Aloísio Falqueto**

Brazil

**Dominique Fameree**

Belgium

**Heng Fan**

China

**Shufang Fan**

USA

**Chi-Tai Fang**

Taiwan

**Hua-Chang Fang**

Taiwan

**Liam Fanning**

Ireland

**Joan Faoagali**

Australia

**Hamed Farag**

Egypt

**Tamer Farag**

USA

**Mansour Farahani**

USA

**Lanfranco Fattorini**

Italy

**David Fedson**

France

**Heinz Feldmann**

USA

**Dan Feng**

China

**Luzhao Feng**

China

**Yaoyu Feng**

China

**Rashida Ferrand**

UK

**Sara Ferrando-Martinez**

Spain

**Catterina Ferreccio**

Chile Chile

**Elisabeth Fichet-Calvet**

Germany

**Richard Fielding**

Hong Kong

**James Fielding**

Australia

**Jordi Figuerola**

Spain

**Katja Fink**

Singapore

**Lydia Finney**

UK

**Alireza Firooz**

Iran

**Marc Fischer**

USA

**Peter Fischer**

USA

**Brian Fisher**

USA

**Jaroslav Flegr**

Czech Republic

**Yvan Fleury**

Switzerland

**Mirembe Florenc**

Uganda

**Mun-Yik Fong**

Malaysia

**Tse-Ling Fong**

USA

**Bradley Ford**

USA

**Nathan Ford**

South Africa

**Ola Forslund**

Sweden

**Silvia Franceschi**

France

**Luigi Franchi**

USA

**Joshua Francis**

Australia

**Suzanna Francis**

UK

**Elisabetta Franco**

Italy

**Luciana Freitas**

Brazil

**Neil French**

Malawi

**Martyn French**

Australia

**Hagen Frickmann**

Germany

**Sk Fridkin**

USA

**N. Deborah Friedman**

Australia

**Gabrielle Froberg**

Sweden

**Holly Frost**

USA

**Chuanxi Fu**

China

**Thomas Fuchs**

Germany

**Jennifer Furin**

USA

**Wieslaw Furmaga**

USA

**Luis Furuya Kanamori**

Australia

**Ana Marisa Fusco Almeida**

Brazil

**Lara Beth Gadkowski**

USA

**Julia Gage**

USA

**Jacques Gaillat**

France

**Patricia Galarza**

Argentina

**Joel Gallant**

USA

**Juan Gálvez-Acebal**

Spain

**Manoj Gambhir**

UK

**Rita Gander**

USA

**Sumanth Gandra**

India

**Maria Garces-Sanchez**

Spain

**Federico Garcia**

Spain

**Melissa Garcia**

USA

**Guillermo Garcia-Effron**

Argentina

**Anthony Garcia-Prats**

South Africa

**Bradley Gardiner**

Australia

**Elvira Garza-Gonzalez**

Mexico

**Alfredo Garzino-Demo**

USA

**Simani Gaseitsiwe**

Botswana

**Roberto Gasparini**

Italy

**Petra Gastmeier**

Germany

**Gaëtan Gavazzi**

France

**Nicholas Geard**

Australia

**Juergen Gebel**

Germany

**Tekebash Gebrekidan**

Ethiopia

**Habip Gedik**

Turkey

**Miguel Gelabert-Gonzalez**

Spain

**Annemieke Geluk**

Netherlands

**Josepa Gené**

Spain

**Brad Gessner**

France

**Fatemeh Ghaffarifar**

Iran

**Souvik Ghosh**

Japan

**Evangelos Giamarellos-Bourboulis**

Greece

**Giovanni Giammanco**

Italy

**Maddalena Giannella**

Italy

**Nicola Gianotti**

Italy

**Michael Gicheru**

Kenya

**Hannah Gideon**

USA

**Phil Giffard**

Australia

**Angel Gil**

Spain

**Jose Pedro Gil**

Sweden

**Rodica Gilca**

Canada

**Carol Gilchrist**

USA

**Christopher Gill**

USA

**Stephen Gillespie**

UK

**Paolo Giorgi Rossi**

Italy

**Raphaële Girard**

France

**Rosina Girones**

Spain

**Marina Giuliano**

Italy

**Olivier Glaizot**

Switzerland

**Awa Gneme**

Burkina Faso

**Nele Goeyvaerts**

Belgium

**Anne Goffard**

France

**Bamenla Goka**

Ghana

**Yoav Golan**

USA

**William Golde**

USA

**Mohamad Goldust**

Iran

**Alicia Gomez Lopez**

Spain

**Ye Gong**

China

**Zuojiong Gong**

China

**Stevan Gonzalez**

USA

**Stuart Gordon**

USA

**Severin Gose**

USA

**Neela Goswami**

USA

**Hiro Goto**

Brazil

**Michihiko Goto**

USA

**Pierre Goussard**

South Africa

**Michele Gouvea**

Brazil

**Susan Graham**

USA

**Lindsay Grant**

USA

**Stephen Graves**

Australia

**James Gray**

UK

**Magdalena Grce**

Croatia

**Alice Green**

USA

**Harleen Grewal**

Norway

**David Griffith**

USA

**Maria Grillet**

Venezuela

**Rolf Groenwold**

Netherlands

**Andreas H. Groll**

Germany

**Robert Gross**

USA

**Itamar Grotto**

Israel

**Peng Guan**

China

**Sajni Gudka**

Australia

**Jeremie Guedj**

France

**Alejandro Gugliucci**

USA

**Marguerite Guiguet**

France

**Nicole Guiso**

France

**Revathi Gunturu**

Kenya

**Ming Guo**

USA

**Shashank Gupta**

USA

**Rodrigo Gurgel-Goncalves**

Brazil

**Carlton Gyles**

Canada

**Abdulrazaq Habib**

Nigeria

**Ivanka Hadji-Petrusheva Meloska**

Macedonia

**Bridget Haire**

Australia

**Judith Melchior Haissman**

Denmark

**Behzad Hajarizadeh**

Australia

**Anita Hällgren**

Sweden

**Jo Halliday**

UK

**Sandra Halonen**

USA

**Toshimitsu Hamasaki**

Japan

**Mark Hamilton**

Canada

**Margaret Hammerschlag**

USA

**Axel Hamprecht**

Germany

**Klaus Hamprecht**

Germany

**Camille Hamula**

USA

**Sonja Hansen**

Germany

**Megumi Hara**

Japan

**Guy Harling**

USA

**Kathryn Harris**

UK

**Patrick Harris**

Australia

**Julie Hart**

Australia

**Marek Hartleb**

Poland

**Stefanie Hartley**

Australia

**Thomas Hartung**

USA

**Rumina Hasan**

Pakistan

**Futoshi Hasebe**

Japan

**Robert-Jan Hassing**

Netherlands

**Timo Hautala**

Finland

**Stephen Hawes**

USA

**Michael Hawkes**

Canada

**Claudia Hawkins**

USA

**Kayoko Hayakawa**

Japan

**Daihai He**

Canada

**Fang He**

Singapore

**Yaqing He**

China

**Pasco Hearn**

Cambodia

**Göran Hedin**

Sweden

**Klaus Hedman**

Finland

**Dagmar Hedrich**

Portugal

**Bethany L Hedt-Gauthier**

USA

**Janneke Heijne**

Netherlands

**Yuji Heike**

Japan

**Sebastian Heimann**

Germany

**Leonhard Held**

Switzerland

**Michelle Helinski**

Uganda

**Thiravat Hemachudha**

Thailand

**Carolyn Hemsley**

UK

**Katherine Henderson**

UK

**Emily Henkle**

USA

**Jean-Michel Heraud**

Madagascar

**Jethro Herberg**

UK

**Melissa Herbst-Kralovetz**

USA

**Trent Herdman**

UK

**Sabine Hermans**

Uganda

**Virginia Hernandez Santiago**

UK

**Victoria Hernando**

Spain

**Björn Herrmann**

Sweden

**Kirsty Hewitt**

UK

**Paul Heyman**

Belgium

**Marylin Hidalgo**

Colombia

**Elizabeth Hilborn**

USA

**Philip Hill**

New Zealand

**Roxane Hillier**

Canada

**Harmanjit Singh Hira **

India

**Keiichi Hiramatsu**

Japan

**Stig Ove Hjelmevoll**

Norway

**Didier Hocquet**

France

**Caspar Hodiamont**

Netherlands

**Martin Hoenigl**

Austria

**Christopher Hoffmann**

USA

**Celia Holland**

Ireland

**David Holland**

USA

**Natasha Holmes**

Australia

**Kam-Lun Ellis Hon**

Hong Kong

**Hitoshi Honda**

USA

**Jan Hontelez**

Netherlands

**Vivian Hope**

UK

**Michael Horberg**

USA

**Juan Pablo Horcajada**

Spain

**Sven Horke**

Germany

**Carolyne Horner**

UK

**Paul Horwood**

Cambodia

**Toni Hospach**

Germany

**Richard Hotchkiss**

USA

**Thomas Allan House**

UK

**Thokozani Hove**

Zimbabwe

**Ching-Hsi Hsiao**

Taiwan

**Ching-Sheng Hsu**

Taiwan

**Alan Yung-Chih Hu**

Taiwan

**Yi Hu**

China

**Yongfei Hu**

China

**Xingdi Hu**

USA

**Angela Huang**

USA

**Chung-Feng Huang**

Taiwan

**Haihui Huang**

China

**Jee-Fu Huang**

Taiwan

**Kai-Wen Huang**

Taiwan

**Yongmei Huang**

USA

**Nils-Olaf Hubner**

Germany

**Claudia Huebner**

Germany

**Linda Hueston**

Australia

**Olivier Huet**

Australia

**Christopher Hull**

USA

**Kristina Hulten**

USA

**Chao-Hung Hung**

Taiwan

**Ivan FN Hung**

Hong Kong

**Christian Hunter**

Namibia

**Shirish Huprikar**

USA

**James Hurley**

Australia

**Dominicus Husada**

Indonesia

**Wilhelmina May Huston**

Australia

**Benedikt Huttner**

Switzerland

**Daniela Huzly**

Germany

**Lars Hviid**

Denmark

**Ji-Yeon Hyeon**

South Korea

**Cristina Ibarra**

Argentina

**Hany Ibrahim**

Egypt

**Ricardo Igreja**

Brazil

**Ute Inegbenebor**

Nigeria

**Teresa Inkster**

UK

**Seth Irish**

USA

**Nahed Ismail**

USA

**Maria Izar**

Brazil

**Mohamed Izham**

Qatar

**Jacques Izopet**

France

**Jan Jacobs**

Belgium

**Samantha Jacobs**

USA

**Raymond Jacobson**

Israel

**Susanne Jacobsson**

Sweden

**Irena Jakopanec**

Norway

**Prince James**

India

**Garth James**

USA

**Hee-Chang Jang**

South Korea

**Won-Jong Jang**

South Korea

**Naveed Janjua**

Pakistan

**Ana Maria Jansen**

Brazil

**Cecile Janssen**

France

**Kari Jarvinen**

Australia

**Marjan Javanbakht**

USA

**Jo Jefferies**

UK

**Wen-Juei Jeng**

Taiwan

**Claire Jenkins**

UK

**Adam Jenney**

Australia

**Jorgen Skov Jensen**

Denmark

**Doosoo Jeon**

South Korea

**Seok Hoon Jeong**

South Korea

**Narayanaperumal Jeyathilakan**

India

**Hua Jiang**

China

**Shibo Jiang**

China

**Zhijun Jie**

China

**Manuel Enrique Jimenez-Mejias**

Spain

**Dong-Yan Jin**

Hong Kong

**Fengyi Jin**

Australia

**Shouguang Jin**

USA

**Chunxia Jing**

China

**Mark Jit**

UK

**Akanitt Jittmittraphap**

Thailand

**Kyung-Wook Jo**

South Korea

**Esaú João**

Brazil

**Tomasz Jodlowski**

USA

**May John**

USA

**Matthew Johnson**

USA

**Nicholas Johnson**

UK

**James Johnston**

Canada

**Barbara Jones**

USA

**Heidi Jones**

USA

**Doug Jones**

USA

**Kelsey Jones**

UK

**Makoto Jones**

USA

**René Jonkers**

Netherlands

**Beenu Joshi**

India

**Martin Joyce-Brady**

USA

**Nicole Juffermans**

Netherlands

**Justin Julander**

USA

**Deven Juneja**

India

**Ji Ye Jung**

South Korea

**Anamarija Jurcev-Savicevic**

Croatia

**Helle Juul-Madsen**

Denmark

**Naoufel Kaabia**

Tunisia

**Achim Kaasch**

Germany

**Adnan Kabaalioglu**

Turkey

**Sushil Kabra**

India

**Edward Kaczmarski**

UK

**Barbara Kahl**

Germany

**Reinhard Kaiser**

Germany

**Siripen Kalayanarooj**

Thailand

**Andre Kalil**

USA

**Jayant Kalpoe**

Netherlands

**Basile Kamgang**

Central African Republic

**Guenter Kampf**

Germany

**Zeina Kanafani**

Lebanon

**Nobuo Kanazawa**

Japan

**Yuko Kanbayashi**

Japan

**Young Ae Kang**

South Korea

**Phyllis Kanki**

USA

**Suman Kanungo**

India

**Peter Kao**

USA

**Nathan Kapata**

Zambia

**Arti Kapil**

India

**Beatrix Kapusinszky**

USA

**Premashis Kar**

India

**Evangelos Karademas**

Greece

**Drosos Karageorgopoulos**

Greece

**Nabil Karah**

Norway

**Petros Karakousis**

USA

**Panagiotis Karanis**

Germany

**Stephen Karpiak**

USA

**Renata Karpiskova**

Czech Republic

**Kumudu Karunaratne**

Sri Lanka Sri Lanka

**Mulya Rahma Karyanti**

Indonesia

**Margaret Kasaro**

Zambia

**Desta Kassa**

Ethiopia

**Elly Katabira**

Uganda

**Achilles Katamba**

Uganda

**David Kateete**

Uganda

**Ioannis Katsarolis**

Greece

**David Katzenstein**

USA

**Sukhbir Kaur**

India

**Kosuke Kawai**

USA

**Takashi Kawamura**

Japan

**Ghazi Kayali**

USA

**Helena Käyhty**

Finland

**Sheila Keating**

USA

**Theodoros Kelesidis**

USA

**Heath Kelly**

Australia

**Paul Kelly**

UK

**Joel Kelso**

Australia

**Brian Kendall**

USA

**Shravan Kethireddy**

USA

**Daniel Kett**

USA

**Olen Kew**

USA

**Pattara Khamrin**

Thailand

**Nitish Khanna**

UK

**Ameneh Khatami**

Australia

**Riad Khatib**

USA

**Ali Khawaja**

Pakistan

**Benson Kidenya**

Tanzania

**Brian Kigozi**

Uganda

**Sandra Kik**

Canada

**Kwang-Sik Kim**

USA

**Hirokazu Kimura**

Japan

**Janni Kinsler**

USA

**Safari Kinung’Hi**

Tanzania

**Agnes Kiragga**

Uganda

**Bruce Kirenga**

Uganda

**Martyn Kirk**

Australia

**Baard Kittang**

Norway

**Eveline Klinkenberg**

Netherlands

**Kerstin Klipstein-Grobusch**

Netherlands

**Bernard Klonjkowski**

France

**Marisa Klopper**

South Africa

**Tejs Klug**

Denmark

**Keith Klugman**

USA

**Vickie Knight**

Australia

**Kwan Soo Ko**

South Korea

**Nobumichi Kobayashi**

Japan

**Marleen Kock**

South Africa

**Robin Köck**

Germany

**Kristian Kofoed**

Denmark

**Utkarsh Kohli**

USA

**Tee Kok Keng**

Malaysia

**Anna Kolecka**

Netherlands

**Ekaterina Kolesanova**

Russia

**Marin H Kollef**

USA

**Ismail Soner Koltas**

Turkey

**Haruki Komatsu**

Japan

**Onn Min Kon**

UK

**Kelika Konda**

USA

**Fabian Kong**

Australia

**Olivier Koole**

UK

**Shyam Kottilil**

USA

**Akiko Kowada**

Japan

**Colleen Kraft**

USA

**Alenka Kraigher**

Slovenia

**Axel Kramer**

Germany

**Florian Krammer**

USA

**Ellen Krautkramer**

Germany

**Carolin Anna-Amalie Kreis**

Germany

**Katrina Kretsinger**

USA

**Marco Aurelio Krieger**

Brazil

**Paula Kriz**

Czech Republic

**Karen Krogfelt**

Denmark

**Christopher Kuaban**

Cameroon

**Adam Kucharski**

UK

**Krystle Kuhs**

USA

**Martin Kulldorff**

USA

**Chandrakanta Kumar**

India

**Rashmi Kumar**

India

**Anil Kumar**

India

**Chien-Min Kung**

Taiwan

**Shajo Kunnath-Velayudhan**

USA

**Preeta Kutty**

USA

**Markku Kuusi**

Finland

**Kin On Kwok**

Hong Kong

**Giuseppe La Torre**

Italy

**Michaela Lackner**

Austria

**Martin Laclaustra**

Spain

**Philippe Lagrange**

France

**Ingrid Laing**

Australia

**Iain Lake**

UK

**András Lakos**

Hungary

**Jimmy Lam**

Hong Kong

**Wai Yip Lam**

Hong Kong

**Paolo Landini**

Italy

**Simone Lanini**

Italy

**Giuseppe Lapadula**

Italy

**Sarah Larney**

Australia

**Anna Lau**

USA

**Eric Lau**

Hong Kong

**Tsai-Ling Lauderdale**

Taiwan

**José Angelo Lauletta Lindoso**

Brazil

**Matthew Laundy**

UK

**Stephen Lawn**

UK

**Andy Lawson**

UK

**Lovett Lawson**

Nigeria

**Jen Layden**

USA

**Eduardo Lazcano-Ponce**

Mexico

**Carlo Lazzaro**

Italy

**Thoa Le**

Viet Nam

**James Le Duc**

USA

**Simon Le Hello**

France

**Vincent Le Moing**

France

**Yann Le Strat**

France

**Jason Leblanc**

Canada

**Sylvie Lecollinet**

France

**Karin Leder**

Australia

**Wen Sen Lee**

Taiwan

**Sai-Cheong Lee**

Taiwan

**Lina Lee**

Taiwan

**Jacob Lee**

South Korea

**Mikyung Lee**

USA

**Seung-Jae Lee**

South Korea

**Jeong-Hoon Lee**

South Korea

**Vernon Lee**

Singapore

**Philippe Lehours**

France

**Eugene Leibovitz**

Israel

**Rasmus Leistner**

Germany

**Tatjana Lejko Zupanc**

Slovenia

**Veerle Lejon**

France

**Magali Lemaitre**

France

**Nadine Lemaitre**

France

**Ligia Lemos**

Brazil

**Oscar Len**

Spain

**Qibin Leng**

China

**Audrey Lenhart**

USA

**Erica Leoni**

Italy

**Olivier Leroy**

France

**Richard Lessells**

South Africa

**Eric Chung Ching Leung**

Hong Kong

**Gilberto Leung**

Hong Kong

**Simon Lévesque**

Canada

**Anna Levin**

Brazil

**Miriam Levy**

Australia

**Sarah Lewis**

USA

**Qihan Li**

China

**Jing Li**

USA

**Linlin Li**

USA

**Qisheng Li**

USA

**Zejun Li**

China

**Wanqing Liao**

China

**Qiuyan Liao**

Hong Kong

**Adamantia Liapikou**

Greece

**Yun-Fan Liaw**

Taiwan

**Viviane Dias Lima**

Canada

**Athena Limnios**

Australia

**Chih-Lin Lin**

Taiwan

**Guangyu Lin**

China

**Hui-Wen Lin**

Taiwan

**Michael Lin**

USA

**Nilton Lincopan**

Brazil

**Lars Lindquist**

Sweden

**Ryan Lindsay**

USA

**Daphne Ling**

Canada

**Li Ling**

China

**Melissa Li-Ng**

USA

**Kristen Little**

USA

**Chun-Jen Liu**

Taiwan

**Sze Hang Kevin Liu**

Hong Kong

**Feng Liu**

USA

**Hsin-Fu Liu**

Taiwan

**Jie Liu**

USA

**Wei Liu**

China

**Zheng Liu**

China

**Carl Llor**

Spain

**Ying-Ru J. Lo**

Switzerland

**Shawn Lockhart**

USA

**Thomas Lodise**

USA

**Michael Loebinger**

UK

**Bettina Löffler**

Germany

**Marc-Arthur Loko**

France

**David Looke**

Australia

**Bruno Silvester Lopes**

UK

**Guillermo Lopez Campos**

Australia

**Luis Eduardo López-Cortés**

Spain

**Mónica López-Lacort**

Spain

**Irene Lorand-Metze**

Brazil

**Jaime Lora-Tamayo**

Spain

**Karen Lorimer**

UK

**Stanka Lotric-Furlan**

Slovenia

**Pierre Loulergue**

France

**Nacer Lounis**

Belgium

**Mona Loutfy**

Canada

**Chris Lowbridge**

Australia

**Chun-Yi Lu**

Taiwan

**Sheng-Nan Lu**

Taiwan

**Li Lu**

USA

**Min-Chi Lu**

Taiwan

**Jean-Christophe Lucet**

France

**Brigid Lucey**

Ireland

**Vivian Luchsinger**

Chile

**Christian Lueck**

Germany

**Grace Lui**

Hong Kong

**Shik Luk**

Hong Kong

**Alexander Lukashev**

UK

**Lucy Lum**

Malaysia

**David Lung**

Hong Kong

**Lixiang Luo**

USA

**Jun Luo**

China

**Irja Lutsar Estonia**

Estonia

**Pascal Lutumba Congo**

Democratic Republic

**Philipp Lutz**

Germany

**Viravarn Luvira**

Thailand

**David Chien Lye**

Singapore

**Hon Ming Ma**

Hong Kong

**Junling Ma**

Canada

**Liying Ma**

China

**Gary Maartens**

South Africa

**Ian Maccormick**

Malawi

**Dorothy Machalek**

Australia

**Margaret Mackinnon**

Kenya

**Richie Madden**

UK

**Dhammika Magana-Arachchi**

Sri Lanka

**Franco Maggiolo**

Italy

**Krisztian Magori**

UK

**Ravi Mahalingam**

USA

**Lumbani Makwakwa**

Malawi

**Fabio Malacarne**

Italy

**Gathsaurie Malavige**

Sri Lanka

**Darren Malinoski**

USA

**Patrick Mallia**

UK

**Kylie-Ann Mallitt**

Australia

**Ryan Malosh**

USA

**Helen Maltezou**

Greece

**Celia Manaia**

Portugal

**Justen Manasa**

South Africa

**Raul Mancilla**

Mexico

**Inacio Mandomando**

Mozambique

**Alireza Mani**

Iran Iran

**Balaji Manicassamy**

USA

**Tobias Manigold**

Switzerland

**Annette Mankertz**

Germany

**Weerawat Manosuthi**

Thailand

**Oriol Manuel**

Switzerland

**Limin Mao**

Australia

**En-Qiang Mao**

China

**Maria Cristina Marazzi**

Italy

**Arnaud Marchant**

Belgium

**Mihai Mares**

Romania

**Vazeille Marie**

France

**Suzanne Marks**

USA

**Caroline Marshall**

Australia

**Laurent Marsollier**

France

**Fernando Marson**

Brazil

**Hanspeter Marti**

Switzerland

**Irene Martin**

Canada

**Belen Martin Agueda**

Spain

**Esteban Martinez**

Spain

**Ignacio Martin-Loeches**

Spain

**Michelle Martinot-Peignoux**

France

**Neil Martinson**

South Africa

**Francisco Marty**

USA

**Marta Massanella**

USA

**Paola Mastrantonio**

Italy

**Sofia Mata-Essayag**

Venezuela

**Ghassan Matar**

USA

**Elizabeth Mathai**

Switzerland

**Barun Mathema**

USA

**Sophie Matheron**

France

**Alice Matimba**

Belgium

**Tadeja Matos**

Slovenia

**Akiko Matsubara**

Japan

**Yasufumi Matsumura**

Japan

**Ana Luiza Mattos-Guaraldi**

Brazil

**Andreas Matussek**

Sweden

**Ryan Maves**

USA

**Harriet Mayanja**

Uganda

**Annarita Mazzariol**

Italy

**John McCauley**

UK

**James McCaw**

Australia

**John McCormick**

Canada

**Jennifer McDanel**

USA

**Joann McDermid**

USA

**Scott McDonald**

UK

**Anita McElroy**

USA

**Lynne McFarland**

USA

**Steve McGloughlin**

Australia

**Chris McGowin**

USA

**Rose McGready**

Thailand

**Bradford McGwire**

USA

**Helen McIlleron**

South Africa

**Robin McKenzie**

USA

**Peter McLaughlin**

USA

**James McMahon**

USA

**Alan McNally**

UK

**Cliodna McNulty**

UK

**Michael McShan**

USA

**Takafira Mduluza**

Zimbabwe

**Rick Meeker**

USA

**Narinder Mehra**

India

**Shaheen Mehtar**

South Africa

**Adam Meijer**

Netherlands

**Chris Meijer**

Netherlands

**Asuncion Mejias**

USA

**Jorge Flavio Mendoza-Rincón**

Mexico

**Francesco Menichetti**

Italy

**Thangam Menon**

India

**Nicolás Merchante**

Spain

**Dominik Mertz**

Canada

**David Mesher**

UK

**David Meya**

Uganda

**Thomas Meyer**

Germany

**Pascal Meylan**

Switzerland

**Patrick Miailhes**

France

**Valeria Micheli**

Italy

**Keren Middelkoop**

South Africa

**Nicholas Midzi**

Zimbabwe

**Gerd Mikus**

Germany

**Matthias Militz**

Germany

**Emma Miller**

Australia

**Olivier Mimoz**

France

**Adrian Mindel**

Australia

**Cezarina Mindru**

USA

**Michele Miraglia Del Giudice**

Italy

**Angelica Miranda**

Brazil

**Satoshi Mitarai**

Japan

**William Mkanta**

USA

**Coceka Mnyani**

South Africa

**Riddhi Modi**

USA

**Rebekah Moehring**

USA

**Harald Moi**

Norway

**Igor Mokrousov**

Russia

**Monica Molano**

Australia

**Solange Moll**

Switzerland

**Marta Mollerach**

Argentina

**Susana Monge**

Spain

**Anna Moniuszko**

Poland

**Emmanuel Montassier**

France

**Kathleen Montone**

USA

**Catherine Moore**

UK

**Catrin Moore**

UK

**Hannah Moore**

Australia

**Luke Moore**

UK

**Luiz Tadeu Moraes Figueiredo**

Brazil

**Beatriz Moreira**

Brazil

**Javier Moreno**

Spain

**Mariza Morgado**

Brazil

**Cory Morin**

USA

**Yoshitomo Morinaga**

Japan

**Ignacio Moriyon**

Spain

**Maria Luisa Moro**

Italy

**Andrew Morris**

Canada

**Brenda Morrow**

South Africa

**Carl Morrow**

South Africa

**Andy Morse**

UK

**Joel Mossong**

Luxembourg

**Kazushi Motomura**

Japan

**Ilaria Motta**

Italy

**Varvara Mouchtouri**

Greece

**Lawrence Moulton**

USA

**Thomas Mourez**

France

**Sabrina John Moyo**

Norway

**Jeffrey Mphahlele**

South Africa

**Judith Mueller**

France

**Mark Mühlau**

Germany

**Khitam Muhsen**

Israel

**Christiaan Mulder**

Netherlands

**Berit Muller-Pebody**

UK

**Jocelyn Mullins**

USA

**Sathish Mundayoor**

India

**Stephen Munga**

Kenya

**Angel G Munoz**

USA

**David Murdoch**

New Zealand

**Sean Murphy**

USA

**Didier Musso French**

Polynesia

**Jukka Mustonen**

Finland

**Nico Mutters**

Germany

**Christina Muzny**

USA

**Dattatraya Muzumdar**

India

**Pauline Mwinzi**

Kenya

**Janet Myers**

USA

**Khin Myint**

Indonesia

**Mathieu Nacher**

French, Guiana

**Anushka Naidoo**

South Africa

**Reshma Naik**

USA

**Cho Naing**

Malaysia

**Clemensia Nakabiito**

Uganda

**Shota Nakamura**

Japan

**Yukihiro Nakanishi**

USA

**Tetsuo Nakayama**

Japan

**Lydia Nakiyingi**

Uganda

**Fernando Nardi**

Italy

**Luisa Nardini**

South Africa

**Masa Narita**

USA

**Kalimuthusamy Natarajaseenivasan**

India

**Dilip Nathwani**

UK

**Roland Nau**

Germany

**Norman Nausch**

UK

**Jabulani Ncayiyana**

South Africa

**Momar Ndao**

Canada

**Patricia Ndhlovu**

UK

**Dieynaba N’Diaye**

France

**Vera Maria Neder Galesi**

Brazil

**Francesco Negro**

Switzerland

**Lisa Nelson**

USA

**Heinrich Neubauer**

Germany

**Sydne Newberry**

USA

**Lisa F.P. Ng**

Singapore

**Simon Ng**

Hong Kong

**Selia Ng’Anjo Phiri**

Norway

**Dung Nguyen Minh**

Viet Nam

**Trang Nguyen Thi Huyen**

Viet Nam

**Luan Nguyen Thien**

Viet Nam

**Emanuele Nicastri**

Italy

**Lindsay Nicolle**

Canada

**Florence Nicot**

France

**Susanne Dam Nielsen**

Denmark

**Albert Nienhaus**

Germany

**Vladyslav Nikolayevskyy**

UK

**Hans Helmut Niller**

Germany

**Nobuyuki Nishikiori**

Philippines

**Yasmine Nivoix**

France

**Philip Norris**

USA

**Alexander Novotny**

Germany

**Marek Nowicki**

USA

**Saad Nseir**

France

**Eric Nuermberger**

USA

**Veronique Nussenblatt**

USA

**Melissa Nyendak**

USA

**Susanne Nylen**

Sweden

**Balthazar Melchior Nyombi**

Norway

**Hipólito Nzwalo**

Portugal

**Stephen Obaro**

USA

**Ponsiano Ocama**

Uganda

**Robinson Ocharo**

Kenya

**Roberta Oconnor**

USA

**John Oeltmann**

USA

**Mark Oette**

Germany

**Tagbo Oguonu**

Nigeria

**Kenji Okada**

Japan

**Peter Okokhere**

Nigeria

**Afiong Oku**

Nigeria

**Oladepo Oladimeji**

Nigeria

**Julian Olalla**

Spain

**Fabiano Oliveira**

USA

**Maura Oliveira**

Brazil

**Maria Isabel Oliveira**

Brazil

**Joseli Oliveira-Ferreira**

Brazil

**Karina Olsen**

Norway

**Ashley Olson**

UK

**Jason Ong**

Australia

**Vincent Onywera**

Kenya

**Wilhelm Oosthuysen**

Germany

**Eline Op De Coul**

Netherlands

**Steven Opal**

USA

**Joseph Oppong**

USA

**Christine Oramasionwu**

USA

**Diane Ordway**

USA

**Eyal Oren**

USA

**Walter Orenstein**

USA

**Andrea Orsi**

Italy

**Miguel O’Ryan**

Chile

**Frank Osei**

Ghana

**Fabio Ostanello**

Italy

**Christian Ostergaard**

Denmark

**Mario Ostrowski**

Canada

**Collins Ouma**

Kenya

**William Kwame Boakye Ansah Owiredu**

Ghana

**Helieh Oz**

USA

**Yasemin Ozsurekci**

Turkey

**Marc Paccalin**

France

**Nesri Padayatchi**

South Africa

**Maria Clara Padoveze**

Brazil

**John Paget**

Netherlands

**Pasquale Pagliano**

Italy

**Dasja Pajkrt**

Netherlands

**Rolando Pajon**

USA

**Bibhuti Bushan Pal**

India

**Pamela Palasanthiran**

Australia

**Palma Palma**

Italy

**Domingo Palmero**

Argentina

**Gaetano Paludetti**

Italy

**Angelo Pan**

Italy

**Akhila Panda**

India

**E Papadogiannakis**

Greece

**Jesse Papenburg**

Canada

**Peter Pappas**

USA

**Dimitrios Paraskevis**

Greece

**Milosz Parczewski**

Poland

**Elena Pariani**

Italy

**Jun Yong Park**

South Korea

**Kyung-Hwa Park**

South Korea

**Steven Park**

USA

**Wan Beom Park**

South Korea

**Young Kil Park**

South Korea

**Michael Parkins**

Canada

**Christopher Parry**

UK

**Charles Parry**

South Africa

**Sally Partridge**

Australia

**Suzan Pas**

Netherlands

**Lisa Pascopella**

USA

**Jotam Pasipanodya**

Zimbabwe

**Sonia Passos**

Brazil

**Nimish Patel**

USA

**Catherine Paugam-Burtz**

France

**John Paul**

UK

**Mical Paul**

Israel

**Federico Pea**

Italy

**Adriana Pedroso**

USA

**Trisha Peel**

Australia

**Martine Peeters**

France

**Eric Walter Pefura-Yone**

Cameroon

**David Pegues**

USA

**Alejandro Peña Monje**

Spain

**Pasi Penttinen**

Finland

**Geraldo M B Pereira**

Brazil

**Julian Perelman**

Portugal

**Ignacio Pérez Valero**

Spain

**Silvia Pérez-Vilar**

Spain

**Robert Perry**

Switzerland

**Nathan Peters**

Canada

**Remco Peters**

South Africa

**Efi Petinaki**

Greece

**William Petri**

USA

**Mariska Petrignani**

Netherlands

**Nicola Petrosillo**

Italy

**Sarah Pett**

UK

**Michael Pfaller**

USA

**Kenneth Pfarr**

Germany

**Yvonne Pfeifer**

Germany

**Tung Phan**

USA

**Benjamin Phelps**

USA

**Julie Philley**

USA

**Nguyen Phuoc Long**

Viet Nam

**Maria Alejandra Picconi**

Argentina

**Chamsai Pientong**

Thailand

**Damas Pierre**

Belgium

**Antonio Pignatari**

Brazil

**Lisa Pineles**

USA

**Jean-Paul Pirnay**

Belgium

**Lionel Piroth**

France

**Tyrone Pitt**

UK

**Gerd Pluschke**

Switzerland

**Antonella Polimeni**

Italy

**Virginia Pomar**

Spain

**Konstantinos Pontikis**

Greece

**Nurith Porat**

Israel

**Angel Porgador**

Israel

**Carolina Porras**

USA

**Frank Post**

UK

**Maarten Postma**

Netherlands

**Pierre Pothier**

France

**Stephanie Pouch**

USA

**Claire Poyart**

France

**Bruno Pozzetto**

France

**Joaquin Prada**

USA

**Tatiana Prado**

Brazil

**John Antony Jude Prakash**

India

**Rosa Prato**

Italy

**Martina Prelog**

Germany

**Rebecca Prevots**

USA

**Alison Price**

Malawi

**Edmond Puca**

Albania

**Andrea Pugliese**

Italy

**Chaturong Putaporntip**

Thailand

**Men-Bao Qian**

China

**Cheng-Feng Qin**

China

**Hongyu Qiu**

Canada

**Liqian Qiu**

China

**M. Ruhul Quddus**

USA

**Petros Rafailidis**

Greece

**Elena Raffetti**

Italy

**Viswanath Ragupathy**

USA

**Juan David Ramirez**

Colombia

**Paula Ramirez**

Spain

**Antonio Ramos**

Spain

**Alberto Ramos**

Brazil

**Meenakshi Rana**

USA

**Harbans S Randhawa**

India

**Nalini Rao**

USA

**Didier Raoult**

France

**Alwyn Rapose**

USA

**Mobeen Rathore**

USA

**Balachandran Ravindran**

India

**Henrik Ravn**

Denmark

**Maria Carla Re**

Italy

**Cathy Reback**

USA

**Helena Rebelo-De-Andrade**

Portugal

**Andrew Redd**

USA

**Elizabeth Reddy**

Tanzania

**David Regan**

Australia

**Gili Regev-Yochay**

Israel

**Antoine Regnault**

France

**Christiane Reichardt**

Germany

**Richard Reid-Smith**

Canada

**Patricia Reis**

Brazil

**William Reisen**

USA

**Lili Ren**

China

**Randall Reves**

USA

**David Rey**

France

**Steven Reynolds**

USA

**Giovanni Rezza**

Italy

**Sofia Ribeiro**

Portugal

**Susanna Ricci**

Italy

**Volker Rickerts**

Germany

**Mark Riddle**

USA

**Maria Helena Rigatto**

Brazil

**Leen Rigouts**

Belgium

**Steven Riley**

UK

**Marius Ringelstein**

Germany

**Giancarlo Ripabelli**

Italy

**Stephen Ritchie**

New Zealand

**Becky Rivoire**

USA

**Jerome Robert**

France

**Jessica Robinson-Papp**

USA

**Monica Robotin**

Australia

**Dan Rockey**

USA

**Joacim Rocklov**

Sweden

**Alejandro Rodriguez**

Spain

**Noris Rodriguez**

Venezuela

**Francisco Rodriguez-Covarrubias**

Mexico

**Carlos Rodriguez-Gallego**

Spain

**Petra Rohrbach**

Canada

**Emmanuel Roilides**

Greece

**Daniela Rojas Castro**

France

**Gabriel Rojas-Ponce**

Peru

**Nigel Rollins**

South Africa

**Lars Rombo**

Sweden

**Elisabeth Root**

USA

**Anne Marie Roque-Afonso**

France

**Bernd Rosenkranz**

Suriname

**Marnie Rosenthal**

USA

**Maria Cristina Rota**

Italy

**Paul Rota**

USA

**Yaron Rotman**

USA

**Coleman Rotstein**

Canada

**Wouter Rottier**

Netherlands

**Francois Rouet**

Gabon

**Perrine Roux**

France

**Sarah Rowland-Jones**

UK

**Yuhua Ruan**

China

**Darren Russell**

Australia

**Fiona Russell**

Australia

**Kevin Russell**

USA

**Silvina Ruvinsky**

Argentina

**Nathan Ryder**

Australia

**Lene Ryom**

Denmark

**Caroline Sabin**

UK

**Ester Cerdeira Sabino**

Brazil

**David Sack**

USA

**Rosemarie Sadsad**

Australia

**Xavier Saelens**

Belgium

**Marco Safadi**

Brazil

**Mahboobeh Safaeian**

USA

**Amar Safdar**

USA

**Akihiko Saitoh**

Japan

**George Sakoulas**

USA

**Nicolas Salez**

France

**Muneeb Salie**

South Africa

**Jorge Salmeron**

Mexico

**Poonam Salotra**

India

**Gilles Salvat**

France

**Mrinal Samanta**

India

**Vittorio Sambri**

Italy

**Georgios Samonis**

Greece

**Jorge Sampaio**

Brazil

**Dhan Samuel**

UK

**Gonzalo Sanchez-Duffhues**

Netherlands

**Matthew Sandbulte**

USA

**Raphael Zozimus Sangeda**

Tanzania

**Marìa De La Paz Santangelo**

Argentina

**Sabine Santibanez**

Germany

**Cristina Santos**

Portugal

**Silvane Santos**

Brazil

**Rajiv Sarkar**

India

**Loredana Sarmati**

Italy

**Michael Satlin**

USA

**Abhay Satoskar**

USA

**Andreas Sauerbrei**

Germany

**A Marion Saville**

Australia

**Robert Sawyer**

USA

**Hugo Sax**

Switzerland

**Sharon Saydah**

USA

**Matthew Schabath**

USA

**Matthew Schaller**

USA

**Dena Schanzer**

Canada

**Frieder Schaumburg**

Germany

**John Schellenberg**

Canada

**Mirte Scherpenisse**

Netherlands

**Oliver Schildgen**

Germany

**Maarten Franciscus Schim Van Der Loeff**

Netherlands

**Werner Schimana**

Germany

**M. Alexander Schmidt**

Germany

**Tina Schmidt**

Germany

**John Schneider**

USA

**Stephen Scholand**

USA

**Kristian Schønning**

Denmark

**Ward Schrooten**

Belgium

**Anna Schultze**

UK

**Jörg Schüpbach**

Switzerland

**Frank Schwab**

Germany

**Eli Schwartz**

Israel

**Jeremy Schwartz**

USA

**Norbert Georg Schwarz**

Germany

**Marin Schweizer**

USA

**Laura Scorzolini**

Italy

**Hyman Scott**

USA

**Anna Seale**

UK

**Holly Seale**

Australia

**Sidy Seck**

Senegal

**John Segreti**

USA

**Philippe Seguin**

France

**Moorine Penninah Sekadde**

Uganda

**Liisa Selin**

USA

**Arlene Sena**

USA

**Lena Serghides**

Canada

**Mireille Serlie**

Netherlands

**Kwonjune Seung**

USA

**Daniel Sexton**

USA

**Otilia Sfetcu**

Sweden

**Marco Sgarbanti**

Italy

**Leigh Anne Shafer**

Canada

**William Shafer**

USA

**Dea Shahinas**

Canada

**Laura Shallcross**

UK

**Masoud Shamaei**

Iran

**Isdore Chola Shamputa**

Canada

**Dennis Shanks**

Australia

**Pei-Lan Shao**

Taiwan

**Suvasini Sharma**

India

**Colin Sharp**

UK

**Gerald Sharp**

USA

**Sunil Shaunak**

UK

**Xingliang Shen**

China

**Adong Shen**

China

**Xin Shen**

China

**Vicky Sheppeard**

Australia

**Jeong Hwan Shin**

South Korea

**Yugo Shobugawa**

Japan

**Joseph Patrick Shott**

USA

**Seyed Davar Siadat**

Iran

**Elopy Sibanda**

Zimbabwe

**Jeanne Sibiude**

France

**Kamran Siddiqi**

UK

**Ilias Siempos**

Greece

**Avelar Silva**

Brazil

**Natal Silva**

Brazil

**Pere Simarro**

Switzerland

**Ian Simms**

UK

**Rj Simonds**

USA

**Leonardo Simonella**

Singapore

**Simon Sin**

Hong Kong

**Mahavir Singh**

Germany

**Sarman Singh**

India

**Sujatha Sistla**

India

**Leung Kei Siu**

Taiwan

**Jelmer Sjollema**

Netherlands

**Anna Skiada**

Greece

**Lemonia Skoura**

Greece

**Laurence Slama**

France

**Vitaly Smelov**

France

**Timo Smieszek**

UK

**Colette Smit**

Netherlands

**Pieter Smit**

UK

**Amber Smith**

USA

**David Smith**

Australia

**M. Kumi Smith**

USA

**Steven G Smith**

UK

**Marlene Smurzynski**

USA

**Davida Smyth**

USA

**Thomas Snelling**

Australia

**Li-Hwei Sng**

Singapore

**Richard Snyder**

USA

**Marcelo Soares**

Brazil

**Rodrigo Soares**

Brazil

**Maja Socan**

Slovenia

**Joseph Solomkin**

USA

**Geoffrey Somi**

Tanzania

**Geetika Sood**

USA

**Alessandro Soria**

Italyv

**Antoni Soriano-Arandes**

Spain

**Giovanni Sotgiu**

Italy

**Alonso Soto**

Peru

**Daniele Souza**

Brazil

**Leigh Sowerby**

Canada

**Akintunde Sowunmi**

Nigeria

**Erica Spackman**

USA

**Michael Spaeder**

USA

**Aris Spathis**

Greece

**C. Wendy Spearman**

South Africa

**David Speers**

Australia

**Iris Spiliopoulou**

Greece

**Gianfranco Spiteri**

Sweden

**Nicola Squillace**

Italy

**Chandrashekhar Sreeramareddy**

Nepal

**Siddharth Sridhar**

Hong Kong

**Vijay Srinivasan**

USA

**Willy Ssengooba**

Uganda

**Rosângela Stadnick Lauth De Almeida Torres**

Brazil

**Philip Stahel**

USA

**Jeffrey Starke**

USA

**Tore W Steen**

Norway

**Pawel Stefanoff**

Poland

**Robert Steffen**

Switzerland

**David Steinberg**

Israel

**Svenja Steinfelder**

Germany

**Christen Rune Stensvold**

Denmark

**Kasia Stepniewska**

UK

**Joanna Stewart**

New Zealand

**O. Colin Stine**

USA

**Tim Stinear**

Australia

**Ellen Stobberingh**

Netherlands

**Lisa Stockdale**

UK

**Nicole Stoesser**

UK

**Jason Stout**

USA

**Jose Stoute**

USA

**Daniel Strickman**

USA

**Jason Stumhofer**

USA

**Dasa Stupica**

Slovenia

**Jonathan D Sugimoto**

USA

**Wataru Sugiura**

Japan

**Sheena Sullivan**

Australia

**Vijay Suppiah**

Australia

**Michael Surette**

Canada

**J. Mark Sutton**

UK

**Troy Sutton**

USA

**Sanjay Swaminathan**

Australia

**Sepehr Tabrizi**

Australia

**Fabienne Tacchini-Cottier**

Switzerland

**Muhamed-Kheir Taha**

France

**Sarah Taimur**

USA

**Samuel Taiwo**

Nigeria

**Tomohiko Takasaki**

Japan

**Howard Takiff**

Venezuela

**Clarence Tam**

UK

**Chor Cheung Tam**

Hong Kong

**Giuseppe Tambussi**

Italy

**Paul Tambyah**

Singapore

**Tina Tan**

USA

**Yi Tan**

USA

**Aaron Tande**

USA

**Neelam Taneja**

India

**Gau-Jun Tang**

Taiwan

**Julian Wei Tze Tang**

Singapore

**Patrick Tang**

Canada

**Weiming Tang**

USA

**Wei Tang**

Japan

**Yi-Wei Tang**

USA

**Giorgio Tarchini**

USA

**Carlo Tascini**

Italy

**Walter Tavares**

Brazil

**Sun Tee Tay**

Malaysia

**Jacqueline Taylor**

Australia

**Lucia Teixeira**

Brazil

**Maria Alice Telles**

Brazil

**Laura Temime**

France

**Chloe Thio**

USA

**Emma Thomas**

Australia

**Luciano Thomazelli**

Brazil

**Kent Thorburn**

UK

**Thorsten Thye**

Germany

**Peng Tian**

USA

**Huai-Yu Tian**

China

**Michael Tildesley**

UK

**Camilla Tincati**

Italy

**Dawn Tladi**

Botswana

**Kelvin To**

Hong Kong

**Catherine Todd**

USA

**Toyin Togun**

Gambia

**Sima Tokajian**

Lebanon

**Steven Tong**

Australia

**Pat Tookey**

UK

**Nuria Torner**

Spain

**Egídio Torrado**

Portugal

**Antoni Torres**

Spain

**Berta Torres**

Spain

**Mehlika Toy**

USA

**Alberto Tozzi**

Italy

**Howard Trachtman**

USA

**Huy A. Tran**

Australia

**David Trees**

USA

**Andrea Trevisan**

Italy

**Priscila Trindade**

Brazil

**Anuradha Tripathy**

India

**Anne Tristan**

France

**Christopher Troeger**

USA

**Cecilia Trucchi**

Italy

**Shaun Truelove**

USA

**Athanassios Tsakris**

Greece

**Chi-Ching Tsang**

Hong Kong

**Georgios Tsivgoulis**

Greece

**Shoji Tsuji**

Japan

**Sarah Tubiana**

France

**Joseph Tucker**

China

**Carrie Tudor**

USA

**Drago Turcinov**

Croatia

**Paul Turner**

Cambodia

**Jane Turton**

UK

**Jimmy Twin**

Australia

**Kerin Tyrrell**

USA

**Georgina Tzanakaki**

Greece

**Akira Ukimura**

Japan

**Christian Ukuru**

Nigeria

**Kingsley Nnanna Ukwaja**

Nigeria

**Anneli Uuskula**

Estonia

**Claude Bernard Uwizeye**

Rwanda

**Yavuz Uyar**

Turkey

**S Vadivelu**

USA

**Viral Vadwai**

India

**Chetana Vaishnavi**

India

**Zoltan Vajo**

Hungary

**Thomas Valentin**

Austria

**Gabriel Vallecillo**

Spain

**Françoise Van Bambeke**

Belgium

**David Van De Vijver**

Netherlands

**Esther Van De Vosse**

Netherlands

**Ingrid Viola Francine Van Den Broek**

Netherlands

**Susan Van Den Hof**

Netherlands

**Akke Van Der Bij**

Netherlands

**Arie Van Der Ende**

Netherlands

**Joke Van Der Giessen**

Netherlands

**Nicoline Van Der Maas**

Netherlands

**Marianne A.B. Van Der Sande**

Netherlands

**Peter Hj Van Der Voort**

Netherlands

**Tjip Van Der Werf**

Netherlands

**Thijs M.A. Van Dongen**

Netherlands

**H Rogier Van Doorn**

Viet Nam

**Rogier Van Doorn**

Viet Nam

**David Van Duin**

USA

**A.M. Van Furth**

Netherlands

**Caroline Van Gemert**

Australia

**Johan Van Griensven**

Belgium

**Rob Van Hest**

Netherlands

**Jakko Van Ingen**

Netherlands

**Maria Van Kerkhove**

UK

**Annelies Van Rie**

USA

**Carla Van Tienen**

Gambia

**Arthur Van Zanten**

Netherlands

**Richard Van Zyl Smit**

South Africa

**Koen Vanden Driessche**

Canada

**Thomas Vanderford**

USA

**Anna H Van’T Hoog**

Netherlands

**Konstantinos Vardakas**

Greece

**Emmanuelle Varon**

France

**Shawn Vasoo**

Singapore

**Tomas Vega Alonso**

Spain

**Irene Veldhuijzen**

Netherlands

**Mario Venditti**

Italy

**Sandro Vento**

Botswana

**Gilles Vergnaud**

France

**Massimiliano Veroux**

Italy

**Sigrid Cjm Vervoort**

Netherlands

**Jaco Verweij**

Netherlands

**Didrik Frimann Vestrheim**

Norway

**Pierluigi Viale**

Italy

**Diego Viasus**

Colombia

**Francesc Vidal**

Spain

**Roberto Vidal**

Chile

**Elvina Viennet**

Australia

**Dhanasekaran Vijaykrishna**

Singapore

**Tereza Villa**

Brazil

**Jean-Louis Vincent**

Belgium

**Leo Visser**

Netherlands

**Matteo Vitali**

Italy

**Astra Vitkauskiene**

Lithuania

**Marco Vitoria**

Switzerland

**Claudia Vitral**

Brazil

**Simonetta Viviani**

Italy

**Lenka Vodstrcil**

Australia

**Petr Volf**

Czech Republic Czech Republic

**Antonio Volpi**

Italy

**Andrea Von Groll**

Brazil

**Nils Von Hentig**

Germany

**Lutz Von Müller**

Germany

**Evridiki Vouloumanou**

Greece

**Tytti Vuorinen**

Finland

**Rosalind Wade**

UK

**Goran Wadell**

Sweden

**Florian Martin Erich Wagenlehner**

Germany

**Hans-Joachim Wagner**

Germany

**Mark Wainberg**

Canada

**Mark Walker**

Australia

**Jack Wallace**

Australia

**Frederic Wallet**

France

**Judd Walson**

USA

**Dalton Wamalwa**

Kenya

**Hongquan Wan**

USA

**Jinliang Wang**

China

**Huan-You Wang**

USA

**Meiliang Wang**

China

**Shih-Min Wang**

Taiwan

**Hanzhong Wang**

China

**Jinfeng Wang**

China

**Wei-Yao Wang**

Taiwan

**Xianjun Wang**

China

**Yujiong Wang**

China

**Sean Wasserman**

South Africa

**Laura Waters**

UK

**Heather Watts**

USA

**Scott Weaver**

USA

**Hsiao L Wei**

Taiwan

**Daniel Weibel**

Netherlands

**Miriam Weinberger**

Israel

**Richard Weiss**

Austria

**Susan Welburn**

UK

**Guilherme Werneck**

Brazil

**David Whiley**

Australia

**Michael White**

UK

**Nicholas John White**

Thailand

**Lucas Wiessing**

Portugal

**Annelies Wilder-Smith**

Singapore

**Barbara Wilhelm**

Canada

**Katalin Wilkinson**

South Africa

**Deborah Williamson**

New Zealand

**Samuel Wilson**

USA

**John Wingard**

USA

**Alan Winston**

UK

**Amy Winter**

USA

**Nathalie Winter**

France

**Linda Wittkop**

France

**Kenneth Witwer**

USA

**Markus Woegerbauer**

Austria

**Cameron Wolfe**

USA

**Petra Fg Wolffs**

Netherlands

**Martin Wolkewitz**

Germany

**Nicole Wolter**

South Africa

**Chia Siong Wong**

Singapore

**Emily Wong**

South Africa

**Frank Wong**

USA

**Ian Wong**

Hong Kong

**Sally Cheuk-Ying Wong**

Hong Kong

**Gary Wong**

China

**Grace Wong**

Hong Kong

**William Chi Wai Wong**

Hong Kong

**Charles Woodrow**

Thailand

**William Worodria**

Uganda

**Colleen Wright**

South Africa

**Dadong Wu**

China

**Lawrence Shih Hsin Wu**

Taiwan

**Shih-Hsiung Wu**

Taiwan

**Zuowei Wu**

USA

**Liyan Xi**

China

**Meng Xiao**

China

**Xianhong Xie**

USA

**Megumi Yamamuro**

Japan

**Hongjiang Yang**

China

**Kun Yang**

China

**Jiyong Yang**

China

**Zhangsheng Yang**

USA

**Tom Yates**

UK

**Barbara Yawn**

USA

**Yazdan Yazdanpanah**

France

**Weimin Ye**

Sweden

**Jae-Joon Yim**

South Korea

**Jiehui Kevin Yin**

Australia

**Louis-Marie Yindom**

UK

**Kai Hang Yiu**

Hong Kong

**Jonny Yokosawa**

Brazil

**Lay-Myint Yoshida**

Japan

**Junichi Yoshida**

Japan

**Hiroshi Yotsuyanagi**

Japan

**Barnaby Young**

Singapore

**Xuejie Yu**

USA

**Hongjie Yu**

China

**Xiaoliang Yuan**

China

**Kwok Yung Yuen**

Hong Kong

**Michele Yuen**

Hong Kong

**Jonghwa Yum**

South Korea

**Yasim Yusof**

Malaysia

**Raffaele Zarrilli**

Italy

**Ingeborg Zehbe**

Canada

**Bernhard Zelger**

Austria

**Roland Zell**

Germany

**Jonathan Zelner**

USA

**Ramzi Zemni**

Tunisia

**Darja Zgur-Bertok**

Slovenia

**Hongliang Zhang**

China

**Lei Zhang**

Australia

**Pan Zhang**

USA

**Furen Zhang**

China

**Guoquan Zhang**

USA

**Hongbo Zhang**

China

**Zhiqiang Zhang**

China

**Zongde Zhang**

China

**Richard Y Zhao**

USA

**Yanping Zhao**

Hong Kong

**Guangming Zhong**

USA

**Dongsheng Zhou**

China

**Jilin Zhou**

USA

**Rong Zhou**

China

**Lei Zhou**

China

**Fengcai Zhu**

China

**Limei Zhu**

China

**Xing-Quan Zhu**

China

**Xun Zhuang**

China

**Panayiotis Ziakas**

USA

**Marya Zilberberg**

USA

**Werner Zimmerli**

Switzerland

**Andrew Zloza**

USA

**Thomas Zoller**

Germany

**Ines Zollner-Schwetz**

Austria

**Zhiyong Zong**

China

**Bruno González Zorn**

Spain

**Huachun Zou**

Australia

**Alexander Zoufaly**

Austria

**Gian Vincenzo Zuccotti**

Italy

